# Opinion: The role of the registered dietitian nutritionist in multiple sclerosis care in the United States

**DOI:** 10.3389/fneur.2023.1068358

**Published:** 2023-02-09

**Authors:** Tyler J. Titcomb, Mona Bostick, Ahmed Z. Obeidat

**Affiliations:** ^1^Department of Internal Medicine, University of Iowa, Iowa City, IA, United States; ^2^Independent Researcher, Greensboro, NC, United States; ^3^Department of Neurology, Medical College of Wisconsin, Milwaukee, WI, United States

**Keywords:** multiple sclerosis, registered dietitian nutritionist, diet, food literacy, opinion

## 1. Introduction

There is great interest in diet among people with multiple sclerosis (MS) (1). Surveys consistently observe that half of people with MS report modifying their diets (2–5). Despite the enthusiasm among people with MS, considerable controversy exists regarding the role of nutritional approaches for MS (6–8). This may be due in part to neurologists reporting feeling inadequately trained in nutrition to provide dietary support (9) and that the research supporting the role for diet in MS is still in preliminary stages (10, 11). For these reasons, people with MS receive little dietary education (12) leading those who are interested in diet to likely utilize internet sources that may not be evidence-based to acquire this information (13).

Several studies indicate that people with MS strongly desire resources and support for including evidence-based dietary guidelines into their treatment and personal wellness plans (14–17). To meet this patient desire, we propose that Registered Dietitian Nutritionists (RDNs) be included on the multidisciplinary care team for people with MS in the United States. In Canada, RDNs are included on the multidisciplinary care team for MS and are considered important members according to a survey of MS health care providers (18). This article provides an overview of RDNs and details some of the key reasons why their inclusion may be beneficial to the care of people with MS.

## 2. Overview of RDNs

### 2.1. Training and licensure

The RDN credential is granted by the Commission on Dietetic Registration (CDR) after completion of master's (formerly baccalaureate) level didactic coursework from an Accreditation Council for Education in Nutrition and Dietetics (ACEND) accredited degree program, supervised practice through a 9–12 months dietetic internship, and passage of the required registration examination for dietitians administered by CDR (19). To maintain the credential, RDNs must complete 75 h of continuing education in 5 years which is documented in and approved by the CDR. RDNs must adhere to the Code of Ethics for the Nutrition and Dietetic Profession (20) and maintain active certification or licensure (in most states) and practice within applicable federal and state laws to practice. These requirements ensure that all RDNs receive training that meets a set of competencies established by the CDR and are how RDNs are distinguished from other “nutritionists” who do not necessarily have the same requirements. Through these distinctions, RDNs are certified to provide medical nutrition therapy to treat specific conditions in clinical practice whereas most other ‘nutritionists' may not have credential requirements or licensure.

### 2.2. The nutrition care process and behavior change

Another means by which RDNs differentiate from other ‘nutritionists' and other healthcare providers is *via* the use of the nutrition care process (NCP), which is a systematic approach to nutrition care that utilizes standardized terminology and is comprised of the following four components: nutrition assessment and reassessment, nutrition diagnosis, nutrition intervention and nutrition monitoring and evaluation (19). This NCP provides a framework for personalized nutrition care that considers a patient's specific goals, needs, and other person-specific factors that can be applied to any individual in any state of health. To facilitate behavior change, RDNs utilize motivational interviewing with goal setting, problem solving, social support, and self-monitoring within the framework of behavior change theory (21).

### 2.3. MS specialization

While the CDR does offer several specialization certifications for RDNs, neither MS nor general neurology are included. However, through the Consortium of MS Centers (CMSC), RDNs can obtain a MS Certified Specialist certification that is offered to all licensed allied healthcare professionals (22).

## 3. Reasons to include RDNs

### 3.1. Avoid pitfalls of online dietary advice

A 2019 scoping review of online web-pages that provide dietary advice targeted toward people with MS found that many specific diets (‘Healthy balanced', Swank low-saturated fat, Wahls Paleolithic, and Best Bet) are recommended online (13), but two recent meta-analyses found that only low-quality evidence supports specialized diets in MS (10, 11). Of these online recommended diets (13), only the “healthy balanced diet”, promoted on webpages by the National MS Society, MS Society of the United Kingdom, and the Mayo Clinic, is based on evidence as these webpages promoted dietary recommendations for increased intake of fruits and vegetables, whole-grains, lean proteins, healthy fats and to restrict/limit added sugar, alcohol, sodium, and ultra-processed foods by the American Heart Association and Dietary Guidelines for Americans (23, 24). Many of the other promoted diets restrict specific foods or nutrients (e.g., grains, dairy) due to the belief that certain dietary antigens, such as gluten and casein, trigger immune-mediated disease activity. Such restrictive diets decrease food variety and increase risk for nutrient deficiencies and disordered eating. For example, the Swank low-saturated fat diet is associated with inadequate intake of vitamins C, A, E, and folate and the Wahls modified Paleolithic diet is associated with inadequate intake of calcium and the vitamins B_12_, D, E, and thiamin among people with MS (25, 26). In addition, sparse evidence suggests that 10% of samples of adults with MS suffer from disordered eating (27–30), which is approximately five times higher than the lifetime prevalence among the general U.S. adult population (31). Given that several surveys show that approximately half of respondents with MS report implementing dietary modifications (2–5), it is possible that many people with MS are at risk for nutrient deficiencies and disordered eating. Through individual screening and promotion of food literacy *via* nutrition education and motivational interviewing, RDNs can support people with MS to avoid these pitfalls by promoting a healthy relationship with food.

### 3.2. Promote food literacy and provide support

Food literacy refers to the tools needed to establish and maintain a lifelong healthy relationship with food (32). Food literacy is highly contextualized as it comprises many person-specific factors including planning and management, selection and acquisition, preparation, and eating (32). In addition, people with physical and cognitive disabilities may require additional support to select, acquire, and prepare healthy food (33). Several studies report that people with MS want additional support to make healthy food choices (14–17). RDNs facilitate behavior change regarding food through motivational interviewing and behavior change theory (21), and are more effective at improving diet quality, glycemic control, cholesterol, and weight management compared to control and other healthcare providers (34, 35). RDNs can help people with MS develop resilience and adaptive strategies regarding food literacy through these patient-centered support strategies to gain knowledge, confidence, and ability in selecting, acquiring, and preparing healthy food.

### 3.3. Screen for food insecurity and malnutrition

Food insecurity is defined as a disruption of food intake due to a lack of resources. People with MS have staggering healthcare costs (36) and tend to exit the workforce earlier compared to people without MS (37), suggesting that food insecurity is possibly an underappreciated consequence of living with MS. A link between disability burden and food insecurity is well documented in several groups without MS; however, this evidence is sparse among people with MS. Importantly, one study observed that people with MS shop for their own food less often compared to healthy controls (38). In addition, several observational studies have observed an inverse association between diet quality and disability burden in MS (38–41), suggesting that people with the greatest disability burden likely have less ability to acquire and prepare healthy foods and likely rely more on convenience foods which tend to be less healthy. In addition, long-term food insecurity increases risk for malnutrition. A 2018 review noted that people with MS have high prevalence of several indicators suggestive of malnutrition including low serum albumin, low BMI, and high prevalence of osteopenia, osteoporosis, vitamin D deficiency, and dysphagia (42). Through validated screening tools (43, 44) and nutrition-focused physical exams (45), RDNs can identify these issues among people with MS (44, 46).

### 3.4. Prevent and manage comorbid conditions

Metabolic risk factors and comorbidities are common among people with MS, and the prevalence is increasing (47). Higher burden of metabolic risk factors and comorbidities is associated with increased MS severity (48–50), brain volume loss (51, 52), and death (53, 54) among people with MS. Low quality diets that are energy-dense, rather than nutrient-dense, increase risk for several metabolic comorbid conditions and risk factors. Observational studies have linked higher diet quality to reduced prevalence of metabolic comorbidities among people with MS (55, 56). Among people without MS, treatments provided by RDNs are effective for weight management and improving lipids, glucose, and blood pressure (57–59); however, no studies have specifically investigated treatment provided by RDNs among people with MS. A few preliminary dietary intervention trials report improvement in biomarkers of metabolic health (60–63) suggesting that treatment provided by RDNs would yield similar favorable outcomes among people with MS.

## 4. Discussion

We propose that including referral for treatment provided by RDNs into the care for people with MS who have interest in diet or a medical justification for the referral, has tremendous upside for patients with minimal risks. To date the majority of preliminary dietary intervention studies link several diets to favorable outcomes among people with MS (11); however, the lack of referral to RDNs has created a situation where people with MS turn to online sources for this information (13). This may predispose people with MS to misinformation that can create distrust with their treating neurologists that may lead to DMT refusal or discontinuation. Without the support of an RDN, the online restrictive diets promoted for MS may cause negative relationships with food or even medical concerns.

While coverage varies between insurance providers, there are some commonalities. For example, clinicians can refer to RDNs and diagnose dietary counseling and surveillance using the billable ICD-10 code Z71.3. This ICD-10 code can be used in conjunction with CPT codes 97802-97804 for medical nutrition therapy or CPT codes 99401-99404 for preventive counseling for risk factor reduction for insurance reimbursement for therapy administered by RDNs. Under the *Affordable Care Act*, diet counseling must be covered by all marketplace health plans without copay or coinsurance for adults at higher risk for chronic disease. Given the high prevalence of comorbid conditions (47), malnutrition (42), and disordered eating (27–30) among people with MS, it is likely that a large proportion may qualify for care, that is covered by insurance, by RDNs who work in a variety of settings including hospitals, clinics, health maintenance organizations, private practice, long-term care facilities, and in community and public health organizations. In addition, treatment by RDNs is cost-effective among people with dyslipidemia and diabetes mellitus (58, 64). For neurologists or other health care providers, who do not have access to in-house RDNs, wishing to refer patients to an RDN, the Academy of Nutrition and Dietetics ‘Find a Nutrition Expert' page (65) or local departments of public health are options to locate an RDN in their local community or *via* telehealth.

## 5. Conclusions

The inclusion of RDNs in multidisciplinary care teams for MS will have other benefits through counseling patients on food:drug interactions (66), increasing the opportunity for research to evaluate the nutritional status and long-term impacts of diet on MS outcomes, and educating other healthcare providers on the benefits of nutrition in the prevention and management of chronic diseases (67, 68). Emerging observational evidence shows that adherence to healthy dietary patterns is associated with several favorable MRI outcomes (69, 70); however, the impact of dietary interventions on MS is remains unclear, albeit promising for patient-reported outcomes (11). This article serves to educate other healthcare providers involved in patient care for people with MS, by outlining several reasons for which people with MS may benefit from care provided by RDNs through promoting health literacy, screening for and preventing food insecurity, malnutrition, and nutrient deficiencies, and for treating and preventing metabolic comorbidities, all issues that are common among people with MS. Inclusion of RDNs in the care for people with MS is justified for several reasons and is a better alternative to the current situation where diet information is not provided in the treatment plan and instead found on online-sources that are mostly not evidence-based. Ultimately, studies are needed to evaluate the impact of treatment provided by RDNs on MS.

## Author contributions

TT wrote the first draft of the manuscript with intellectual input from MB and AO. All authors read, revised, and approved of the final version.
